# Supplementary material for: Occurrence of Antimicrobial Resistance in Canine and Feline Bacterial Pathogens in Germany under the Impact of the TÄHAV Amendment in 2018
Source: Antibiotics (Basel). 2023 Jul 15;12(7):1193. doi: 10.3390/antibiotics12071193 (PMC10376885; doi:10.3390/antibiotics12071193)
Supplement: Supplementary file 1 [file antibiotics-12-01193-s001.zip › antibiotics-2486718-supplementary.pdf]

Descriptive analysis of examined bacterial species for agents influenced by the TÄHAV from 2015 to 2021

Table S1: ***Staphylococcus pseudintermedius***

**year**	**2015**		**2016**		**2017**		**2018**		**2019**		**2020**		**2021**	
Frequency (F) Percent (P)	F	P	F	P	F	P	F	P	F	P	F	P	F	P
total	366	100.0	422	100.0	409	100.0	533	100.0	495	100.0	452	100.0	479	100.0
dog	355	97.0	407	96.4	394	96.3	510	95.7	469	94.7	428	94.7	453	94.6
cat	11	3.0	15	3.6	15	3.7	23	4.3	26	5.3	24	5.3	26	5.4
Penicillin G	286	78.1	329	78.0	296	72.4	387	72.6	352	71.1	331	73.2	322	67.2
Ampicillin	256	72.1/355	275	67.6/407	268	68.0/394	370	72.5/510	334	71.2/469	314	73.4/428	286	63.1/453
Amoxicillin Clavulanic acid	43	12.0/357	40	10.0/399	57	14.8/385	69	13.4/514	55	11.5/480	59	13.6/435	57	12.2/469
Cefovecin	59	16.1	50	11.8	58	14.2	62	11.6	53	10.7	59	13.1	59	12.3
Enrofloxacin	45	12.3	34	8.1	53	13.0	46	8.6	35	7.1	26	5.8	34	7.1

Table S2: ***Staphylococcus felis***

**year**	**2015**		**2016**		**2017**		**2018**		**2019**		**2020**		**2021**	
Frequency (F) Percent (P)	F	P	F	P	F	P	F	P	F	P	F	P	F	P
total	47	100.0	65	100.0	66	100.0	122	100.0	131	100.0	128	100.0	109	100.0
dog	1	2.1	0	0	1	1.5	3	2.5	0	0	1	0.8	1	0.9
cat	46	97.9	65	100.0	65	98.5	119	97.5	131	100.0	127	99.2	108	99.1
Penicillin G	34	72.3	32	49.2	26	39.4	44	36.1	46	35.1	39	30.5	36	33.0
Ampicillin	27	58.7/46	28	43.1	25	38.5/65	41	34.5/119	46	35.1	38	29.9	34	31.5
Amoxicillin Clavulanic acid	11	23.4	5	7.7	10	15.2	6	4.9	6	4.6	8	6.3	2	1.8
Cefovecin	13	27.7	4	6.2	9	13.6	5	4.1	6	4.6	8	6.3	2	1.8
Enrofloxacin	5	10.6	2	3.1	9	13.6	3	2.5	2	1.5	5	3.9	2	1.8

Table S3: ***Staphylococcus aureus***

**year**	**2015**		**2016**		**2017**		**2018**		**2019**		**2020**		**2021**	
Frequency (F) Percent (P)	F	P	F	P	F	P	F	P	F	P	F	P	F	P
total	59	100.0	62	100.0	49	100.0	75	100.0	75	100.0	68	100.0	76	100.0
dog	42	71.2	41	66.1	31	63.3	36	48.0	42	56.0	31	45.6	33	43.4
cat	17	28.8	21	33.9	18	36.7	39	52.0	33	44.0	37	54.4	43	56.6
Penicillin G	52	88.1	53	85.5	31	63.3	53	70.7	50	66.7	51	75.0	58	76.3
Ampicillin canin	34	81.0/42	33	80.5/41	20	64.5/31	30	83.3/36	30	71.4/42	25	80.6/31	23	69.7/33
Ampicillin felin	12	70.6/17	19	90.5/21	11	61.1/18	23	59.0/39	20	60.6/33	26	70.3/37	32	74.4/43
Amoxicillin Clavulanic acid	24	41.4/58	30	48.4/62	4	8.2/49	14	19.2/73	19	25.7/74	17	25.0/68	9	12.0/75
Cefovecin	25	42.4	31	50.0	5	10.2	15	20.0	19	25.3	15	22.1	9	11.8
Enrofloxacin	12	20.3	19	30.6	5	10.2	8	10.7	7	9.3	9	13.2	3	3.9

Table S4: canine ***E. coli*** of the urinary tract

**year**	**2015**		**2016**		**2017**		**2018**		**2019**		**2020**		**2021**	
Frequency (F) Percent (P)	F	P	F	P	F	P	F	P	F	P	F	P	F	P
dog	37	100.0	44	100.0	46	100.0	55	100.0	59	100.0	57	100.0	34	100.0
Ampicillin	9	24.3	12	27.3	18	39.1	17	30.9	12	20.3	13	23.2	9	26.5
Amoxicillin Clavulanic acid	2	5.4	1	2.3	5	10.9	6	10.9	3	5.1	6	10.7	3	8.8
Cefovecin	4	10.8	10	22.7	9	19.6	7	12.7	3	5.1	3	5.4	1	2.9
Enrofloxacin	8	21.6	9	20.5	10	21.7	9	16.4	4	6.8	4	7.1	2	5.9

Table S5: ***E. coli*** of soft and skin tissue

**year**	**2015**		**2016**		**2017**		**2018**		**2019**		**2020**		**2021**	
Frequency (F)<br>Percent (P)	F	P	F	P	F	P	F	P	F	P	F	P	F	P
total	179	100.0	219	100.0	187	100.0	349	100.0	316	100.0	307	100.0	305	100.0
dog	148	82.8	187	85.4	148	79.1	262	75.1	239	75.6	233	75.9	240	78.7
cat	31	17.2	32	14.6	39	20.9	87	24.9	77	24.4	74	24.1	65	21.3
Cefovecin	50	27.8	74	33.8	26	13.9	76	21.8	25	7.9	39	12.7	35	11.5
Enrofloxacin	30	16.7	26	11.9	26	13.9	27	7.7	25	7.9	16	5.2	28	9.2

Table S6: *Proteus mirabilis*

year	2015		2016		2017		2018		2019		2020		2021	
Frequency (F)<br>Percent (P)	F	P	F	P	F	P	F	P	F	P	F	P	F	P
total	51	100.0	60	100.g0	53	100.0	90	100.0	83	100.0	67	100.0	73	100.0
dog	48	94.1	57	95.0	49	92.5	82	91.1	79	95.2	60	89.6	67	91.8
cat	3	5.9	3	5.0	4	7.5	8	8.9	4	4.8	7	10.4	6	8.2
Cefovecin	12	23.5	21	35.0	6	11.3	20	22.2	12	14.5	9	13.4	11	15.1
Enrofloxacin	5	9.8	7	11.7	8	15.1	8	8.9	8	9.6	6	9.0	4	5.5

Table S7: *Klebsiella* spp.

**year**	**2015**		**2016**		**2017**		**2018**		**2019**		**2020**		**2021**	
Frequency (F)<br>Percent (P)	F	P	F	P	F	P	F	P	F	P	F	P	F	P
total	18	100.0	22	100.0	19	100.0	40	100.0	37	100.0	41	100.0	29	100.0
dog	17	94.4	22	100.0	16	84.2	36	90.0	33	89.2	34	82.9	26	89.7
cat	1	5.6	0	0	3	15.8	4	10.0	4	10.8	7	17.1	3	10.3
Cefovecin	3	16.7	7	31.8	2	10.5	4	10.0	7	18.9	4	9.8	6	20.7
Enrofloxacin	0	0	1	4.5	2	10.5	0	0	0	0	3	7.3	1	3.4

Table S8: ***Pasteurella multocida***

**year**	**2015**		**2016**		**2017**		**2018**		**2019**		**2020**		**2021**	
Frequency (F) Percent (P)	F	P	F	P	F	P	F	P	F	P	F	P	F	P
total	63	100.0	73	100.0	91	100.0	188	100.0	189	100.0	186	100.0	174	100.0
dog	11	17.5	26	35.6	28	30.8	26	13.8	27	14.3	23	12.4	33	19.0
cat	52	82.5	47	64.4	63	69.2	162	86.2	162	85.7	163	87.6	141	81.0
Ampicillin	0	0	3	4.1	2	2.2	13	6.9	2	1.1	4	2.2	3	1.7
Amoxicillin Clavulanic acid	0	0	1	1.4	1	1.1	1	0.5	0	0	1	0.5	0	0
Cefovecin	3	4.8	3	4.3	1	1.1	12	6.5	2	1.1	2	1.1	2	1.1
Enrofloxacin	0	0	0	0	1	1.1	1	0.5	1	0.5	0	0	2	1.1

Table S9: *β-hemolytic Streptococcus* spp.

year	2015		2016		2017		2018		2019		2020		2021	
Frequency (F) Percent (P)	F	P	F	P	F	P	F	P	F	P	F	P	F	P
total	137	100.0	146	100.0	139	100.0	232	100.0	215	100.0	202	100.0	156	100.0
dog	125	91.2	143	97.9	125	89.9	204	87.9	184	85.6	174	86.1	131	84.0
cat	12	8.8	3	2.1	14	10.1	28	12.1	31	14.4	28	13.9	25	16.0
Penicillin G	0	0	0	0	0	0	0	0	0	0	0	0	0	0
Ampicillin	0	0	0	0	0	0	0	0	0	0	0	0	0	0
Amoxicillin Clavulanic acid	0	0	0	0	0	0	0	0	0	0	0	0	0	0
Cefovecin	0	0	0	0	0	0	0	0	0	0	0	0	0	0
Enrofloxacin	0	0	0	0	0	0	0	0	0	0	2	1.0	0	0
